# Green-Synthesized *Chaenomeles speciosa*–Derived Carbon Quantum Dots with Blue Fluorescence and Selective Pro-Apoptotic Effects in Cancer Cells

**DOI:** 10.1007/s10895-026-04771-y

**Published:** 2026-04-29

**Authors:** Zeynep Betül Sarı, Abidin Gümrükçüoğlu, Emine İncilay Torunoğlu, Muhammet Emin Sarı, Erdi Can Aytar

**Affiliations:** 1 Faculty of Medicine, Medical Biology, Ankara Yıldırım Beyazıt University, Ankara, 06010 Türkiye; 2 Medicinal-Aromatic Plants Application and Research Center, Artvin Çoruh University, Artvin, 08000 Türkiye; 3 Faculty of Medicine, Department of Medical Biochemistry, Necmettin Erbakan University, Konya, 42090 Türkiye; 4 Faculty of Medicine, Department of Medical Biology, Necmettin Erbakan University, Konya, 42090 Türkiye; 5 Faculty of Agriculture, Department of Horticulture, Usak University, Uşak, 64200 Türkiye

**Keywords:** Apoptosis, Cytotoxicity, FTIR, Wound healing assay, XPS, *Chaenomeles speciosa*

## Abstract

Carbon nanodots were synthesized from *Chaenomeles speciosa* flowers and comprehensively characterized. UV–Vis analysis revealed a broad absorption band from 200 to 400 nm, attributed to π→π* (200–250 nm) and n→π* (250–350 nm) transitions, while fluorescence under 365 nm excitation showed bright blue emission. HRTEM images indicated spherical nanoparticles forming clustered aggregates, with lattice fringes exhibiting interplanar spacings of 0.091–0.093 nm, confirming a partially graphitic structure. XRD analysis showed a broad amorphous baseline in the 10–40° 2θ range, indicating low crystallinity. FTIR and XPS analyses identified abundant surface –OH, C = O, and C–O groups, with elemental composition of C 68.28%, O 23.03%, N 6.70%, S 0.40%, and Si 1.59%, resulting in a C/O ratio of 2.97. *Chaenomeles speciosa* quantum dots (CS-CQDs) exhibited dose-dependent cytotoxicity in MDA-MB-231 cells (viability: 70% at 2.5 mg/mL, 60% at 5 mg/mL) and HeLa cells (approximately 50% at 2.5–5 mg/mL), with minimal effects on hTERT-HME1 cells (viability greater than 85% at 0.6 mg/mL). Apoptosis increased from 25% to 55% in MDA-MB-231, from 24% to 65% in HeLa, and from 5.9% to 25% in HME1, with negligible necrosis. Gene expression analyses showed NF-κB upregulation (3-fold in MDA-MB-231; 6-fold in HeLa), BAX and BCL-2 upregulation in HeLa (approximately 2.5-fold), and downregulation of HIF1A, IL-6, MMP2, and MMP9. Wound healing assays indicated reduced migration in MDA-MB-231 (44% to 21%), increased migration in HeLa (18.7% to 30%), and minimal change in HME1 (73.5% to 71.5%). These results demonstrate that CS-CQDs possess favorable optical and structural properties and selectively induce apoptosis in cancer cells, with minimal impact on non-cancerous cells, highlighting their potential as targeted anticancer nanomaterials.

**Supplementary Information:**

The online version contains supplementary material available at 10.1007/s10895-026-04771-y.

## Introduction

Cervical cancer and breast cancer are among the most prevalent malignancies in women worldwide, representing significant causes of cancer-related mortality. Cervical cancer arises from the abnormal proliferation of epithelial cells in the uterine cervix, with 80% of cases occurring in underdeveloped regions, and is the fourth most common cancer in women, following breast and colorectal [1]. Surgical intervention is recommended for early-stage (IA-IIA) cervical cancer, while chemoradiotherapy and radiotherapy are generally employed for advanced-stage (IIB-IVA) cases. However, conventional treatments are limited by low drug efficacy, lack of specificity, adverse side effects, and multidrug resistance, resulting in reduced long-term effectiveness [2]. Breast cancer, the most diagnosed cancer globally, reported over 2.05 million new cases in 2018, and its incidence is projected to rise by more than 46% by 2040 [3, 4]. Triple-negative breast cancer (TNBC), an aggressive subtype, is characterized by rapid progression, high metastatic potential, and the absence of effective targeted therapies, leaving treatment largely dependent on surgery, radiotherapy, and chemotherapy [3–5]. Consequently, there is a critical need for optimized and targeted therapeutic strategies. Cellulose-based materials, owing to their superior chemical, physical, and thermal properties, as well as their environmentally friendly and adaptable nature, enable the development of nanoscale supercapacitors, energy systems, sensors, and composite materials [6]. Nanotechnology-based drug delivery systems offer promising solutions by enabling organ-specific targeting, preserving drug bioactivity, promoting selective accumulation, reducing toxicity, and maintaining therapeutic concentrations for extended periods, potentially improving treatment efficacy and minimizing recurrence [7–10].

Natural plants are considered an important resource for maintaining human health and combating diseases [11]. *Chaenomeles speciosa* (Sweet) Nakai, a deciduous shrub of the Rosaceae, can tolerate semi-shaded, cold, and arid conditions and is mainly distributed in temperate regions such as China, Korea, and Japan [12–14]. Its fruit is nutrient-rich, containing sugars, proteins, vitamins, and pectin, and has been used clinically to alleviate conditions such as colds, asthma, hepatitis, and rheumatoid arthritis. The sap of *C. speciosa* can promote wound healing on ulcerated skin, and certain components extracted from the plant are used in the development of drugs for rare myofibrillar myopathy, recognized as orphan drugs by the FDA [15]. With its aesthetic appearance and bright colors, *C. speciosa* is also valued as an ornamental plant, making it a versatile species with medicinal, nutritional, and decorative applications [16].

In this context, the development of biocompatible and environmentally friendly nanocomposites has emerged as an important research area for both antimicrobial and anticancer applications [17]. In this study, the flower of *C. speciosa* was used as a precursor for the synthesis of carbon quantum dots through a simple hydrothermal process. The successful synthesis was confirmed by a comprehensive analysis of morphology and surface properties using TEM, FTIR, XRD, UV-Vis, and other techniques. Therefore, it is of great importance to rapidly discover and investigate natural resources with minimal side effects for drug development. In this context, the aim of the present study was to investigate the antitumor, apoptosis-inducing, wound-healing effects, and gene expression of Q-dots obtained from *C. speciosa* via hydrothermal methods on the HeLa cell line (cervical cancer) and the MDA-MB-231 cell line (breast cancer).

## Experimental Section

### Plant-Based Hydrothermal Carbon Quantum Dots (CQDs)

The flower material of *C. speciosa* was dried at room temperature in an environment protected from moisture and direct sunlight. The dried plant material was ground using a mechanical blender and passed through a 200-mesh sieve to obtain a fine powder. The prepared plant powder (2 g) was transferred into a 100 mL breaker, and 70 mL of deionized water, corresponding to approximately 70% of the reactor volume, was added. The mixture was subjected to ultrasonic treatment for 30 min to ensure homogeneous dispersion.

Subsequently, the homogeneous mixture was transferred into a 100 mL Teflon-lined hydrothermal reactor, which was then placed in an electric oven. The hydrothermal carbonization process was carried out at 200 °C for 12 h under a closed system. After completion of the process, the reactor was allowed to cool to room temperature. The resulting yellow–brown solution was filtered sequentially using Whatman filter paper followed by a 0.2 μm membrane filter [18]. The morphology and particle size of the synthesized CS-CQDs were evaluated from HRTEM micrographs using ImageJ software (NIH, USA); individual particle diameters were measured from representative images to assess the size distribution of the nanoparticles.

Various analytical techniques were employed for the characterization of carbon dots (CQDs). The applied analytical methods and the purpose of each technique in the characterization process are summarized in Table 1.

**Table 1:** Methods used in the characterisation of CQDs and the purpose of these methods

Method	Intended use
XPS	To determine the surface elemental composition of materials
HRTEM measurements	Determining whether materials are crystalline or amorphous at the nanoscale
XRD	To obtain information about the crystalline structure of materials
FTIR	Determining the types of chemical bonds in the structure of materials
UV-Vis spectroscopy	Determination of the light absorption properties of materials

### Hydrothermal Preparation of *Chaenomeles speciosa* Carbon Quantum Dots (CS-CQDs)

The aqueous extract *Chaenomele speciosa* carbon quantum dots (CS-CQDs) were dissolved in distilled water (28 mg/mL), filtered through a 0.25 μm syringe filter, and diluted in serum-free DMEM to prepare a 5 mg/mL working solution, which was freshly prepared before each experiment for cell treatment.

### Cell Culture

MDA-MB-231 (triple-negative breast cancer) and HeLa (human cervical cancer cell line) cell lines were utilized as cancer models, while hTERT-HME1 (human mammary epithelial cell line) served as a non-cancerous cell line control. Cells were maintained in DMEM (Diagnovum, Netherlands) supplemented with 10% FBS (Gibco, USA) and 1% penicillin–streptomycin (Gibco, USA) at 37 °C in a humidified 5% CO₂ incubator.

### Cell Viability Assay

MTT assay was used to evaluate the cytotoxicity of CS-CQDs in HeLa and MDA-MB-231 cells [19]. Cells were seeded in 96-well plates (1 × 10⁴/well), allowed to attach for 24 h, and treated with 0.3–2.5 mg/mL extract for 48 h. After incubation, MTT solution (20 µL; 1 mg/mL) was added for 3 h, formazan crystals were dissolved in 150 µL DMSO, and absorbance was measured at 570 nm. IC₅₀ values were calculated using GraphPad Prism (v8.4). Non-tumorigenic HME-1 cells were exposed to these IC₅₀ concentrations for subsequent viability, apoptosis, and morphological assays.

### Quantitative Real-Time PCR (qRT-PCR)

To analyze apoptosis-related gene expression, total RNA was extracted from treated and control samples using an RNA isolation kit (Bio Basic, Canada) according to the manufacturer’s protocol [20]. cDNA was synthesized with the OneScript cDNA Synthesis Kit (ABM, Canada), and quantitative PCR was carried out using SYBR Green PCR Master Mix on the Roche LightCycler 480 II platform. The primer sequences used are presented in Table 2.

**Table 2:** Primer sequences of genes analyzed by quantitative real-time PCR

Gene	Primer sequence
Bcl-2	F 5′-GTGGATGACTGAGTACCTGAAC-3′, R 5′-GAGACAGCCAGGAGAAATCAA-3′
Bax	F 5′-GGAGCTGCAGAGGATGATG-3′, R 5′-GGCCTTGAGCACCAGTTT-3′
HIF1A	F 5′‑CCCTAACGTGTTATCTGTCGCT‑3′, R 5′‑TCTCAAGAATTTGCGTTAGGGC‑3′
NFKB1	F 5′‑GTGAACCCATTCCTCACCCA‑3′, R 5′‑GCGCTGAATCTTTGCTATGG‑3′
GAPDH (reference gene)	F 5′-GAAGGTGAAGGTCGGAGT-3′, R 5′-GAAGATGGTGATGGGATTTC-3′

Relative gene expression was calculated using the 2–ΔΔCT method, with GAPDH as the reference gene. The expression levels in treated groups were compared directly to those in untreated controls

### Wound Healing Assay

Cell migration was evaluated by a wound healing assay [21]. Cells (1 × 10⁴/well) were seeded in 96-well plates and grown to confluency for 24 h. A scratch was created using a sterile pipette tip, after which the cells were either treated with the test compound or supplied with fresh medium (control). Images of the wound area were captured at 0 and 24 h using an inverted microscope (4×), and wound closure was quantified using ImageJ software. The percentage of cell migration was calculated for each condition using the following formula:

$$Migration\;(\%)\;=\;\lbrack(Wound\;area\;at\;0\;h\:-\:Wound\;area\;at\;24\;h)\;/\;Wound\;area\;at\;0\;h\rbrack\;\times\;100$$

This assay was performed independently for each cell line to evaluate the effect of the treatment on cell motility.

### Apoptosis Assay

Apoptosis was assessed using an Annexin V-FITC/PI Detection Kit (Elabscience, China) according to the manufacturer’s instructions. Cells were seeded in 24-well plates, treated with IC₅₀ concentrations of the compound for 48 h, harvested, and washed with PBS. Following staining with Annexin V-FITC and PI for 10–15 min in the dark, the cells were examined under a fluorescence microscope (Olympus BX51, Japan). For analysis, approximately 100 cells were evaluated per condition. Live cells appeared unstained or faintly green, early apoptotic cells exhibited green fluorescence (Annexin V⁺/PI⁻), and late apoptotic or necrotic cells showed both green and red fluorescence signals (Annexin V⁺/PI⁺). The apoptotic index was calculated as the proportion of Annexin V-positive cells relative to the total number of cells [22].

### Statistical Analysis

Data are presented as mean ± standard deviation (SD) from three independent experiments. Statistical analyses were performed using GraphPad Prism (version 8.0; GraphPad Software, USA). Comparisons between two groups (e.g., treated vs. control) were conducted using Student’s t-test. For RT–qPCR, relative gene expression was calculated using the 2^−ΔΔCt method, and statistical testing was performed on ΔCt values. A p-value < 0.05 was considered statistically significant. Significance levels are indicated as follows: *p* < 0.05, **p < 0.01, *p < 0.001, and ***p < 0.0001.

## Results and Discussion

### UV–Vis and Fluorescence Characterization of Carbon Nanodot

**Fig. 1:**
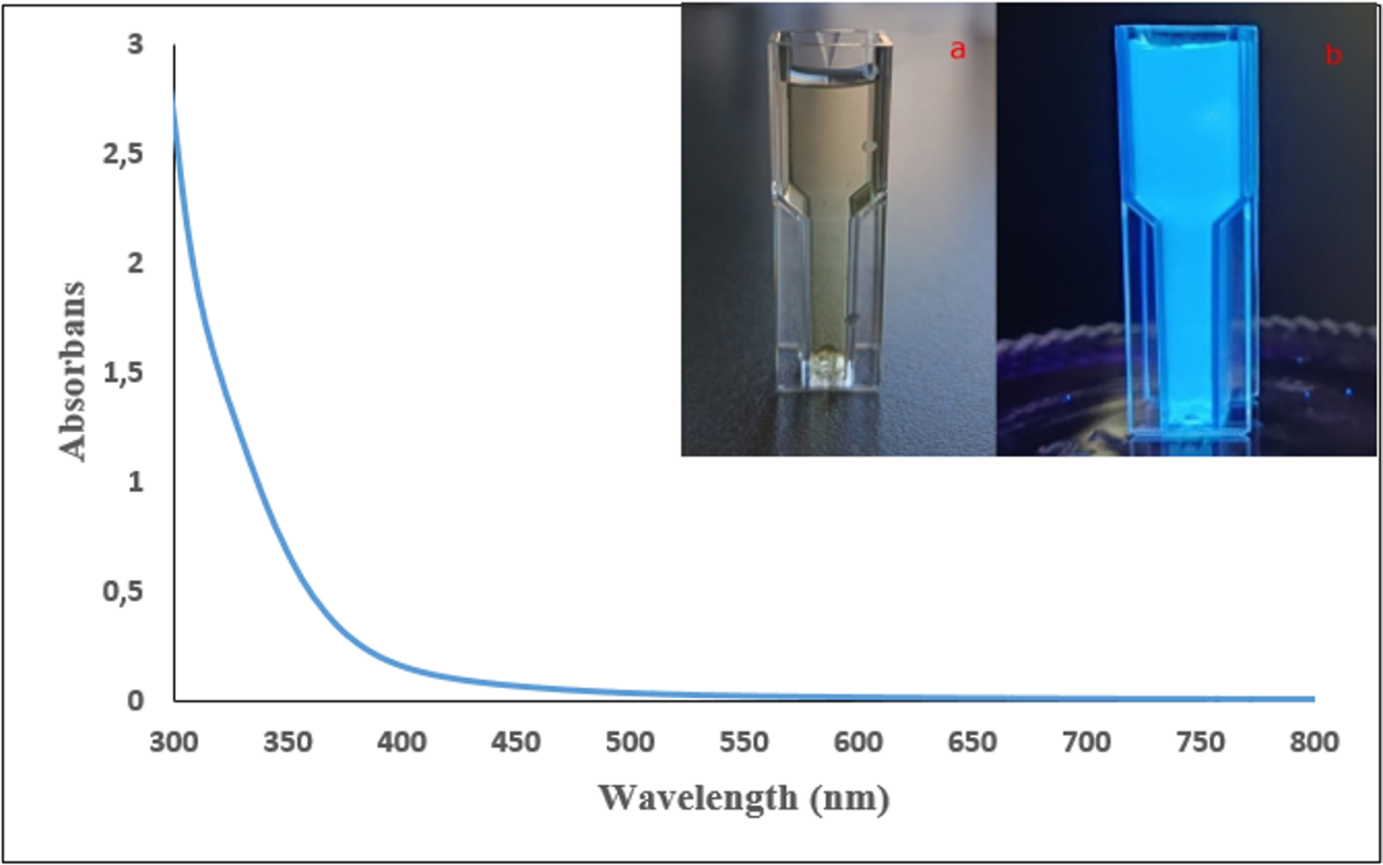
Photoluminescent properties of carbon nanodots

The optical properties of the synthesized CS-CQDs were investigated using UV–Vis and fluorescence spectroscopy. A 2 mL aliquot of the stock aqueous CS-CQDs solution was diluted to a final volume of 4 mL with deionized water, resulting in a 1:1 dilution. The UV–Vis absorption spectrum of the diluted solution is presented in Fig. 1 and provides important insights into the optical behavior of the carbon nanodots.

The carbon nanodots exhibit a broad absorption band starting at approximately 200 nm and extending into the visible region. The strong absorption observed in the 200–250 nm range is attributed to π→π electronic transitions of aromatic C = C bonds. In addition, surface C = O functional groups contribute to absorption in the 250–350 nm region through n→π* transitions. The overlap of these two electronic transitions leads to the formation of a single, broad, and continuous absorption band in the 200–400 nm range [23–26]. This characteristic confirms the presence of a π-electron system within the carbon nanodot structure and indicates successful surface functionalization. The observed absorption features are consistent with previously reported literature and arise from π-electronic transitions involving sp²-hybridized carbon atoms and heteroatom-containing surface groups [27].

Under normal visible light, the carbon nanodots exhibit a homogeneous appearance with no noticeable luminescence (Fig. 1a). In contrast, when irradiated at a wavelength of 365 nm, a bright blue fluorescence is clearly observed (Fig. 1b). This observation demonstrates that the CS-CQDs absorb ultraviolet light and emit visible light, confirming the characteristic photoluminescent behavior of carbon nanodots synthesized from a single plant precursor. Overall, these results verify the successful synthesis of CS-CQDs and the expected development of their optical properties.

Al-Ghamdi et al. [28] synthesized ZnO nanoparticles using *Moringa oleifera* leaf extracts and demonstrated that these nanostructures are promising materials for various applications. In contrast to that study, the present work investigates the anticancer activities of CS-CQDs-based nanoparticles.

The biological activities of NiO-CMC-Dcar nanocomposites are generally attributed in the literature to enhanced ROS generation associated with oxygen vacancies [29]. In contrast, the cytotoxic effects of the CS-CQDS nanostructures developed in this study may be related to surface functional groups and cellular interaction mechanisms. Furthermore, while NiO-CMC-Dcar structures exhibit pronounced biocidal activity, CS-CQDS nanostructures demonstrate cell type-dependent cytotoxic responses. These findings indicate that the two nanomaterial systems differ in both composition and underlying mechanisms of biological activity.

### Structural Characterization of Carbon Quantum Dots by HRTEM

**Fig. 2:**
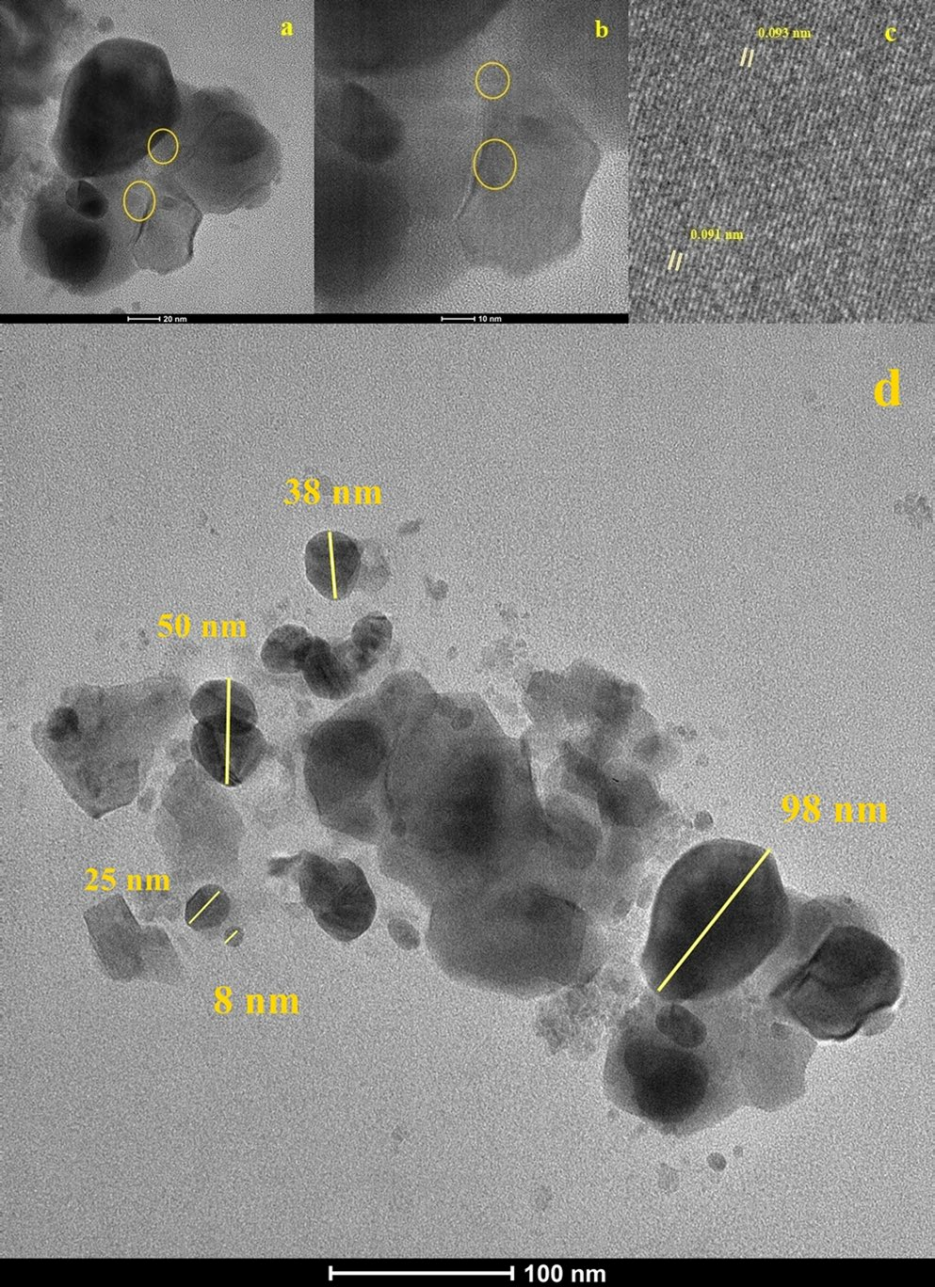
HRTEM images of carbon nanodots

In order to investigate the crystalline structure of the carbon nanodots, high-resolution transmission electron microscopy (HRTEM) images were obtained at different magnification levels (Fig. 2a and c). The low-magnification image (Fig. 2a) shows that the approximately spherical nanoparticles are in close contact with each other, forming clustered structures. In the aggregate regions marked with yellow circles, the particles are observed to overlap and form stacked arrangements (scale bar: 20 nm).

The medium-magnification image (Fig. 2b) provides a detailed view of the aggregation behavior. In the region highlighted by the yellow circle, a heterogeneous distribution of amorphous areas and well-ordered crystalline layers can be clearly observed. Periodically arranged lattice fringes (crystal plane lines) demonstrate the crystalline nature of the carbon nanodots (scale bar: 10 nm).

The high-resolution image (Fig. 2C) allows for a detailed atomic-scale analysis of the crystalline region. The lattice fringes highlighted by the red box are clearly visible, revealing the periodic arrangement of graphitic layer planes. The interplanar spacing, determined by direct measurement, is observed to range between 0.093 and 0.091 nm. These lattice spacing values indicate that the carbon nanodots possess nanoscale dimensions and a partially graphitic structure, fully consistent with the broad amorphous baseline observed in XRD and the high degree of oxygen functionalization identified by FTIR.

Figure 2D presents a wide-field HRTEM image used for particle size analysis (scale bar: 100 nm). Individual particle diameters were measured using ImageJ software, revealing a heterogeneous size distribution ranging from 8 to 98 nm. This wide size range reflects the aggregation-prone nature of hydrothermally synthesized carbon dots, consistent with the clustered morphology observed in Fig. 2A and B.

### X-ray Diffraction Study of Carbon Quantum Dots

**Fig. 3:**
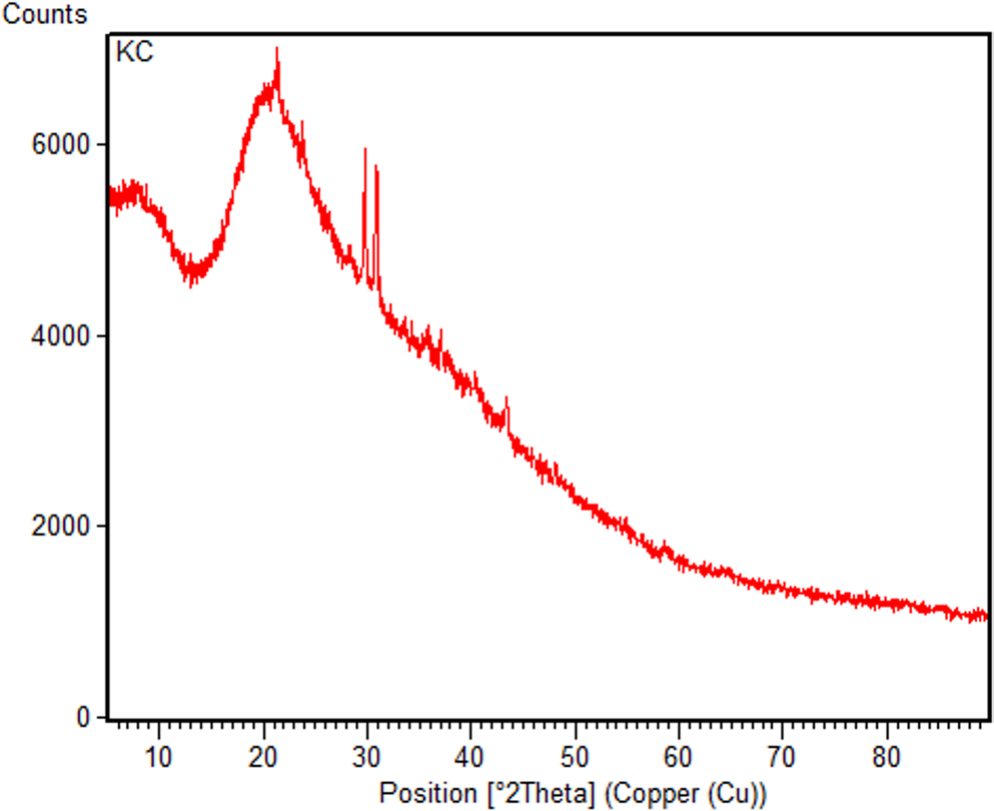
XRD diffractogram of carbon quantum Dots

X-ray Diffraction (XRD) analysis characterizes the crystalline structure of the synthesized carbon nanodots (Fig. 3). Measurements were performed over the 2θ range of 10–80°. A broad and intense baseline is observed in the 10–40° region of the diffractogram. This broad background is a strong indication that the carbon nanodots are predominantly amorphous in nature.

Upon detailed examination of the XRD peaks, d-spacing values of 4.09 Å at 2θ = 21.5°, 3.01 Å at 2θ = 30°, and 2.88 Å at 2θ = 31° are obtained. However, these peaks appear very weak in the diffractogram. The broad and low-intensity peak in the 2θ ≈ 20–25° region originates from the amorphous carbon background.

The low intensity of the crystalline peaks together with the broad amorphous background indicates that the carbon nanodots are of nanoscale dimensions and exhibit low crystallinity. This structural feature is typical for carbon nanodots at the nanometer scale. The most prominent characteristic of the diffractogram is the dominance of the amorphous structure with only weak crystalline reflections.

HRTEM analysis revealed lattice spacing distances of 0.093 nm and 0.091 nm. These values are significantly smaller than the d-spacing of the graphite (002) plane, which is 0.335 nm. The amorphous structure indicated by XRD and the weak crystalline features observed by HRTEM together confirm that the synthesized carbon nanodots possess an amorphous–crystalline hybrid structure.

### FTIR Analysis of Carbon Quantum Dots

**Fig. 4:**
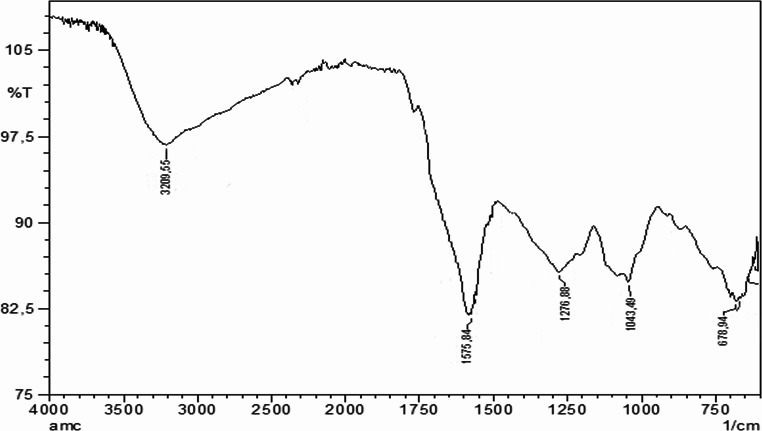
FTIR spectrum of carbon quantum dots

Fourier Transform Infrared Spectroscopy (FTIR) analysis reveals the functional groups present on the surface of the synthesized carbon nanodots (Fig. 4). Five main absorption peaks are observed in the FTIR spectrum, each corresponding to specific chemical bonds.

The broad absorption band at 3209.5 cm⁻¹ corresponds to O–H stretching vibrations. This broad and high-intensity peak indicates a high concentration of –OH groups on the surface of the carbon nanodots. The peak at 1575.84 cm⁻¹ is attributed to a combination of C = O carbonyl and C = C aromatic stretching vibrations.

The peak at 1276.88 cm⁻¹ represents C–O stretching vibrations, while the peak at 1043.49 cm⁻¹ corresponds to C–O stretching vibrations associated with ether (C–O–C) and alcohol (C–OH) groups. The peak at 648.94 cm⁻¹ indicates C–C bending vibrations.

The FTIR results demonstrate that the surface of the synthesized carbon nanodots is coated with a variety of oxygen-containing functional groups, particularly –OH, C = O, and C–O. This surface modification imparts a hydrophilic character to the carbon nanodots and ensures their high solubility in water.

### XPS Analysis of Carbon Quantum Dots

**Fig. 5:**
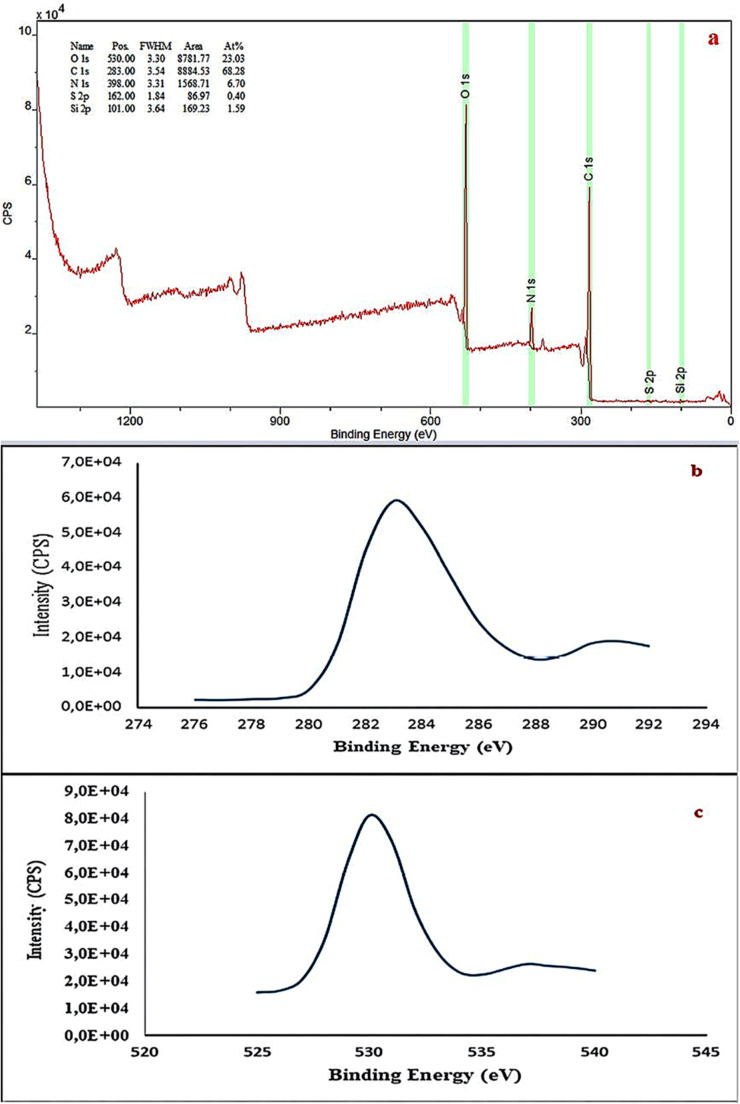
XPS of carbon quantum dots

X-ray Photoelectron Spectroscopy (XPS) analysis provides a detailed characterization of the surface elemental composition and chemical states of the synthesized carbon nanodots (Fig. 5). The XPS survey spectrum reveals the presence of five elements. Carbon (C) accounts for 68.28%, oxygen (O) for 23.03%, nitrogen (N) for 6.70%, sulfur (S 2p) for 0.40%, and silicon (Si) for 1.59%.

The presence of silicon (1.59%) originates from the silicate glass substrate on which the film was formed during the sample preparation process. Since XPS is a surface-sensitive technique with an analysis depth of approximately 3–5 nm, the influence of the glass substrate is observed. The relative intensity of the Si peak reflects the thickness and structure of the carbon nanodot film. The detection of Si in the XPS spectrum confirms the correctness of the sample preparation methodology.

Nitrogen (6.70%) in the synthesized carbon nanodots originates from two sources. The first source is the phenolic compounds, amino acids, and nitrogen-containing plant metabolites naturally present in the *Chaenomeles speciosa* precursor material. The second source arises from nitrogen-containing functional groups formed during the decomposition of the biological material throughout the hydrothermal carbonization process. This nitrogen is primarily bonded to the surface of the carbon nanodots in the form of –N–C = O (amide) structures, –NH₂ amino groups, and pyridine-like nitrogen-containing heteroaromatic structures.

The *C. speciosa* flower contains a high number of phenolic compounds, including flavonoids, tannins, and phenolic acids. During the hydrothermal carbonization process, the aromatic rings of these phenolic compounds form the carbon framework, while the associated amino groups and nitrogen-containing structures serve as nitrogen sources. Consequently, the detected nitrogen provides evidence that nitrogen naturally originating from the plant source has been successfully integrated into the surface of the carbon nanodots through the synthesis process.

The high-resolution C 1s spectrum (Fig. 5b) shows that the main peak center is located at 283 eV. This spectrum indicates that carbon atoms exist in different chemical environments. Deconvolution analysis identifies sp²-hybridized graphitic-like carbon (C = C), sp³-hybridized aliphatic carbon (C–C), C–O bonds, C = O carbonyl groups, and O–C = O carboxyl groups. The high-resolution O 1s spectrum (Fig. 5c) exhibits a peak center at 530 eV and consists of two main components. C–O bonds (ether and phenolic groups) are located at approximately 531–532 eV, while C = O bonds (carbonyl and carboxyl groups) appear at approximately 533 eV. The presence of phenolic –OH groups is confirmed by the intensity of the C–O component [30].

According to the atomic concentration analysis, the C/O ratio is calculated as 68.28/23.03 = 2.97. This high C/O ratio indicates the carbon-rich nature of the carbon nanodots. The XPS results demonstrate that the synthesized carbon nanodots consist of a core structured by sp² and sp³ carbon and a surface coated with oxygen- and nitrogen-containing functional groups derived from plant phenolic compounds. The Si signal originates from the silicate glass substrate and confirms the success of the synthesis.

### Cytotoxic Effects of CS-CQDs on Cancer and Non-Cancerous Cells

To evaluate the cytotoxic effects of CS-CQDs, MTT assays were performed on three different cell lines: MDA-MB-231, HeLa and hTERT-HME1. The results demonstrated that CS-CQDs exhibited differential cytotoxic effects across these cell lines.

**Fig. 6:**
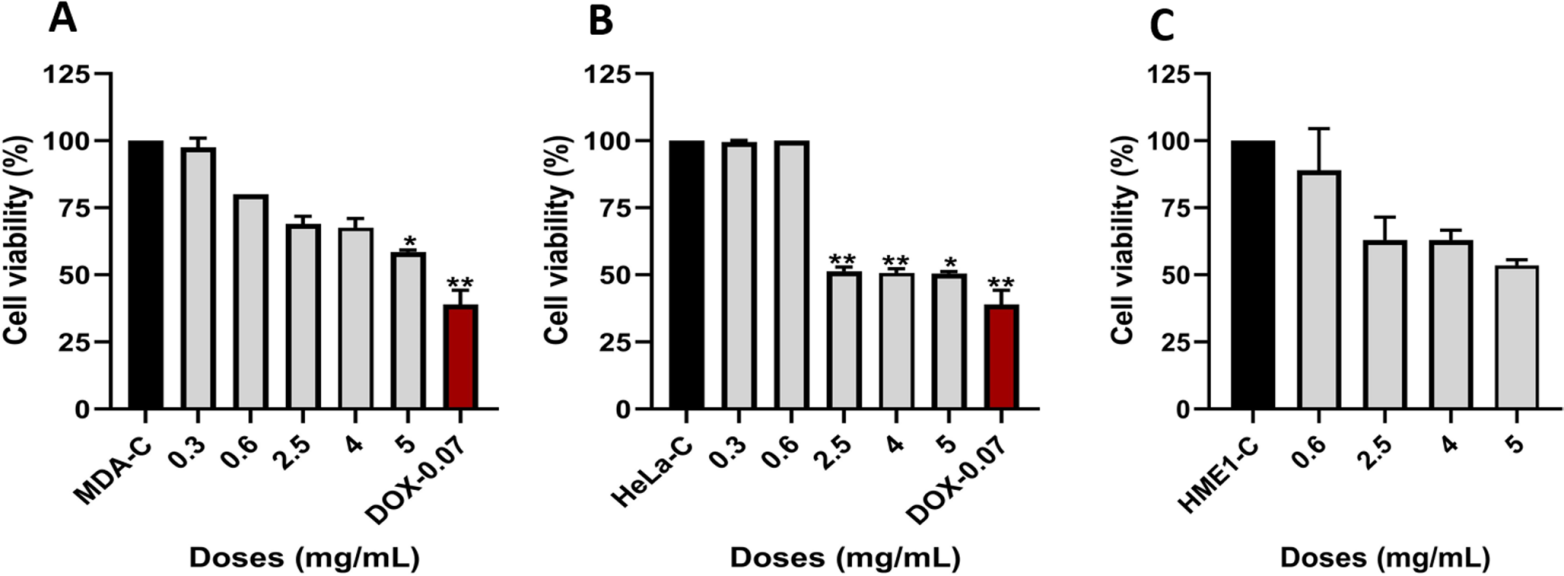
Effect of CS-CQDs on cell viability in MDA-MB-231, HeLa, and HME-1 cells. The impact of CS-CQDs on cell viability was assessed using the MTT assay. (**A**) MDA-MB-231, (**B**) HeLa, and (**C**) HME-1 cells were treated with increasing concentrations of CS-CQDs. MDA-C, HeLa-C, and HME1-C represent non-treated control groups. Doxorubicin (DOX), a known anticancer agent, was used as a positive control. Data are presented as percentage of viable cells relative to controls. Statistical significance: *p < 0.05, **p < 0.01 versus corresponding control group

In MDA-MB-231 cells, a dose-dependent decrease in cell viability was observed with increasing concentrations of CS-CQDs. Cell viability declined to approximately 70% at 2.5 mg/mL, 67% at 4 mg/mL, and 60% at 5 mg/mL (*p < 0.05). Doxorubicin (DOX, 0.07 mg/mL), used as a positive control, showed the most pronounced effect, reducing viability to around 35% (**p < 0.01). The IC₅₀ value of CS-CQDS for MDA-MB-231 cells was calculated to be approximately 5 mg/mL (Fig. 6A).

In HeLa cells, although statistically significant cytotoxicity was observed at 2.5, 4, and 5 mg/mL (*p < 0.05, **p < 0.01), cell viability remained relatively constant at around 50% across these doses. This suggests a plateau effect, where increasing the dose did not further enhance cytotoxicity. The maximum effect of CS-CQDs in HeLa cells was still weaker than that of DOX. Lower concentrations (0.3 and 0.6 mg/mL) had no significant impact on viability. The IC₅₀ value was also estimated at approximately 5 mg/mL (Fig. 6B).

In non-cancerous hTERT-HME1 cells, minimal toxicity was observed at 0.6 mg/mL, with cell viability above 85%. A gradual reduction in viability occurred at 2.5, 4, and 5 mg/mL, with the highest dose reducing viability to ~ 55%, indicating moderate dose-dependent cytotoxicity in normal cells (Fig. 6C).

Overall, MTT assay results revealed that CS exerted a clear dose-dependent cytotoxic effect in MDA-MB-231 cells, while in HeLa and HME1 cells, a plateau effect was observed at higher concentrations. This plateau may be attributed to the saturation of intracellular targets or differences in cellular uptake or response mechanisms. The comparable sensitivity of HeLa and HME1 cells suggests that CS-CQDs ’s cytotoxicity may not be highly selective between cancerous and non-cancerous epithelial cells in certain contexts.-CS-CQDs

### Morphological Evaluation of the Cells

**Fig. 7:**
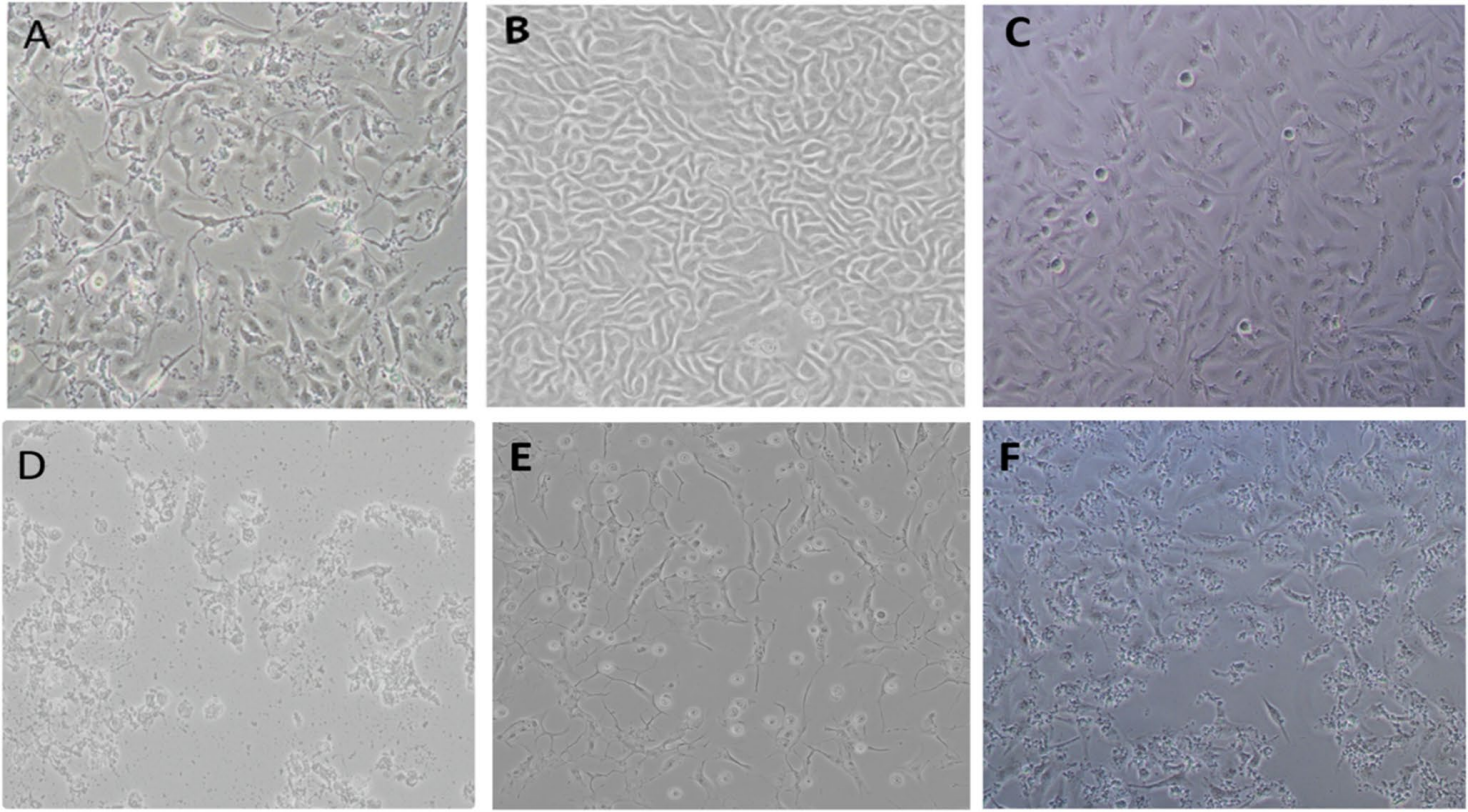
Morphological changes in MDA-MB-231, HeLa, and HME-1 cells. Representative images of MDA-MB-231 (**A**), HeLa (**B**), and HME-1 (**C**) cells in untreated control groups, and MDA-MB-231 (**D**), HeLa (**E**), and HME-1 (**F**) cells following CS-CQDs treatment. Cells were observed under a light microscope at 10× magnification

Significant morphological changes were observed in MDA-MB-231, HeLa, and HME-1 cells following CS-CQDs treatment using light microscopy at 10× magnification (Fig. 7). Under control conditions, all three cell lines exhibited their characteristic morphology: MDA-MB-231 cells showed an elongated, spindle-like appearance typical of mesenchymal breast cancer cells (Fig. 7A); HeLa cells displayed a tightly packed, cobblestone-like epithelial pattern (Fig. 7B); and HME-1 cells, representing non-cancerous mammary epithelial cells, formed an organized monolayer with uniform elongated shapes (Fig. 7C). In contrast, CS-CQDs -treated cells exhibited notable alterations. MDA-MB-231 cells appeared more rounded and detached, with decreased confluency, indicating loss of adhesion and potential cytotoxicity (Fig. 7D). HeLa cells showed disrupted cell–cell junctions and membrane blebbing, suggestive of cellular stress or death (Fig. 7E). While HME-1 cells also exhibited some morphological changes, such as partial detachment and reduced density, the extent of disruption was markedly less compared to the cancer cell lines (Fig. 7F).

These findings indicate that CS-CQDs induces pronounced morphological alterations particularly in cancerous cells, suggesting a possible selective cytotoxic effect on malignant versus healthy cells.

### Gene Expression Profiles Show Cell-Type Specific Regulation After CS-CQDs Treatment

**Fig. 8:**
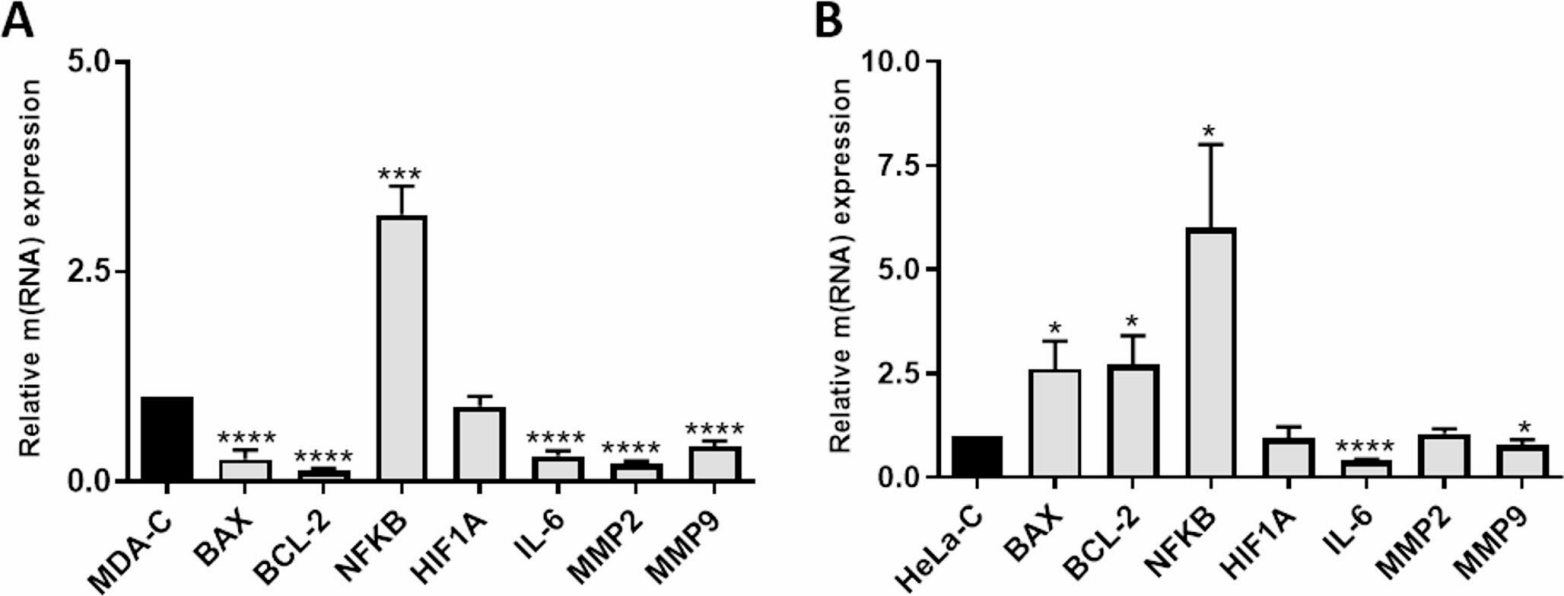
Relative gene expression levels in MDA-MB-231 and HeLa cells following CS-CQDs treatment. (**A**) MDA-MB-231 cells and (**B**) HeLa cells were treated with the IC₅₀ concentration of AÇM, and mRNA expression levels of apoptosis-related (BAX, BCL-2), inflammation-related (NF-κB, IL-6), hypoxia-related (HIF1A), and metastasis-related (MMP2, MMP9) genes were analyzed by qPCR. Untreated cells (MDA-C and HeLa-C) were used as control groups and set as reference (1-fold). Data are presented as mean ± SD. Statistical significance is indicated as *p < 0.05; **p < 0.01; ***p < 0.001; ****p < 0.0001

Following CS-CQDS treatment in MDA cells, a significant alteration in gene expression profiles was observed. Notably, NF-κB expression was markedly upregulated, showing approximately a three-fold increase compared to the control group (***p < 0.001). In contrast, all other analyzed genes showed a decrease in expression levels; however, this downregulation was statistically significant for all genes except HIF1A. These findings suggest a selective activation of NF-κB signaling in response to CS-CQDs, while at the same time reducing the expression of the other genes (Fig. 8A).

In HeLa cells, gene expression analysis following CS-CQDs treatment revealed a significant upregulation of BAX, BCL-2, and NF-κB, with approximately 2.5-fold, 2.5-fold, and 6-fold increases, respectively, compared to the control group (*p < 0.05). Conversely, the expressions of HIF1A, IL-6, MMP2 and MMP9 were decreased following treatment. Among these, the downregulation of IL-6 and MMP9 was statistically significant, with IL-6 showing the highest level of significance (****p < 0.0001) and MMP9 showing a moderate but significant reduction (*p < 0.05) (Fig. 8B).

### CS-CQDs Treatment Modulates Cell Migration in a Cell-Type-Specific Manner

**Fig. 9:**
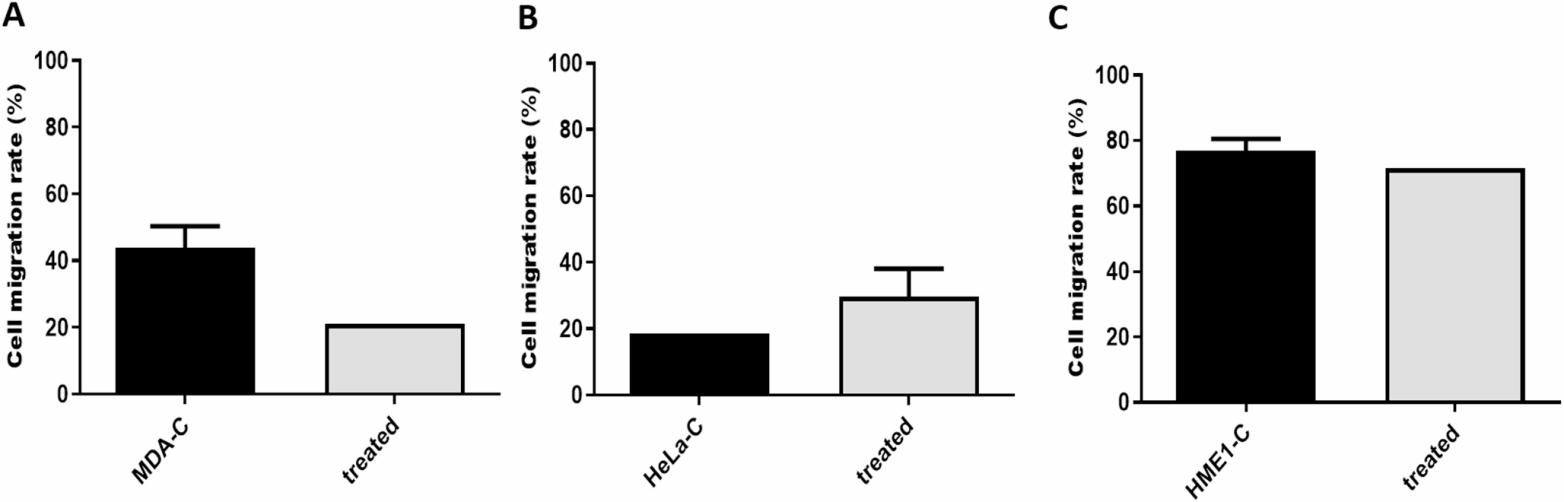
Effects of CS-CQDs on cell migration in MDA-MB-231, HeLa, and HME-1 cell lines. Cell migration rates (%) were measured using the wound healing assay in untreated (MDA-C, HeLa-C, HME1-C) and CS-CQDS -treated groups

The effects of CS-CQDs treatment on cell migration were evaluated in MDA-MB-231, HeLa, and HME-1 cell lines using the wound healing assay. In MDA-MB-231 cells, the migration rate decreased from approximately 44% in the control group to 21% following CS-CQDs treatment (Fig. 9A). Conversely, in HeLa cells, CS-CQDs treatment slightly increased the migration rate from 18.7% to about 30% (Fig. 9B). In the non-cancerous HME-1 cell line, migration was only minimally affected, decreasing from 73.5% to 71.5% (Fig. 9C). However, none of these changes reached statistical significance (*p* > 0.05).

Although the results were not statistically significant, the marked reduction in migration observed in MDA-MB-231 cells supports our hypothesis that CS-CQDs may suppress cell motility in aggressive cancer cell types. These preliminary findings suggest that CS-CQDs has the potential to impair migration in a cell-type-specific manner, particularly in highly migratory cells.

### CS-CQDs Selectively Induces Apoptosis in Cancer Cells Without Affecting Non-Cancerous Cells

**Fig. 10:**
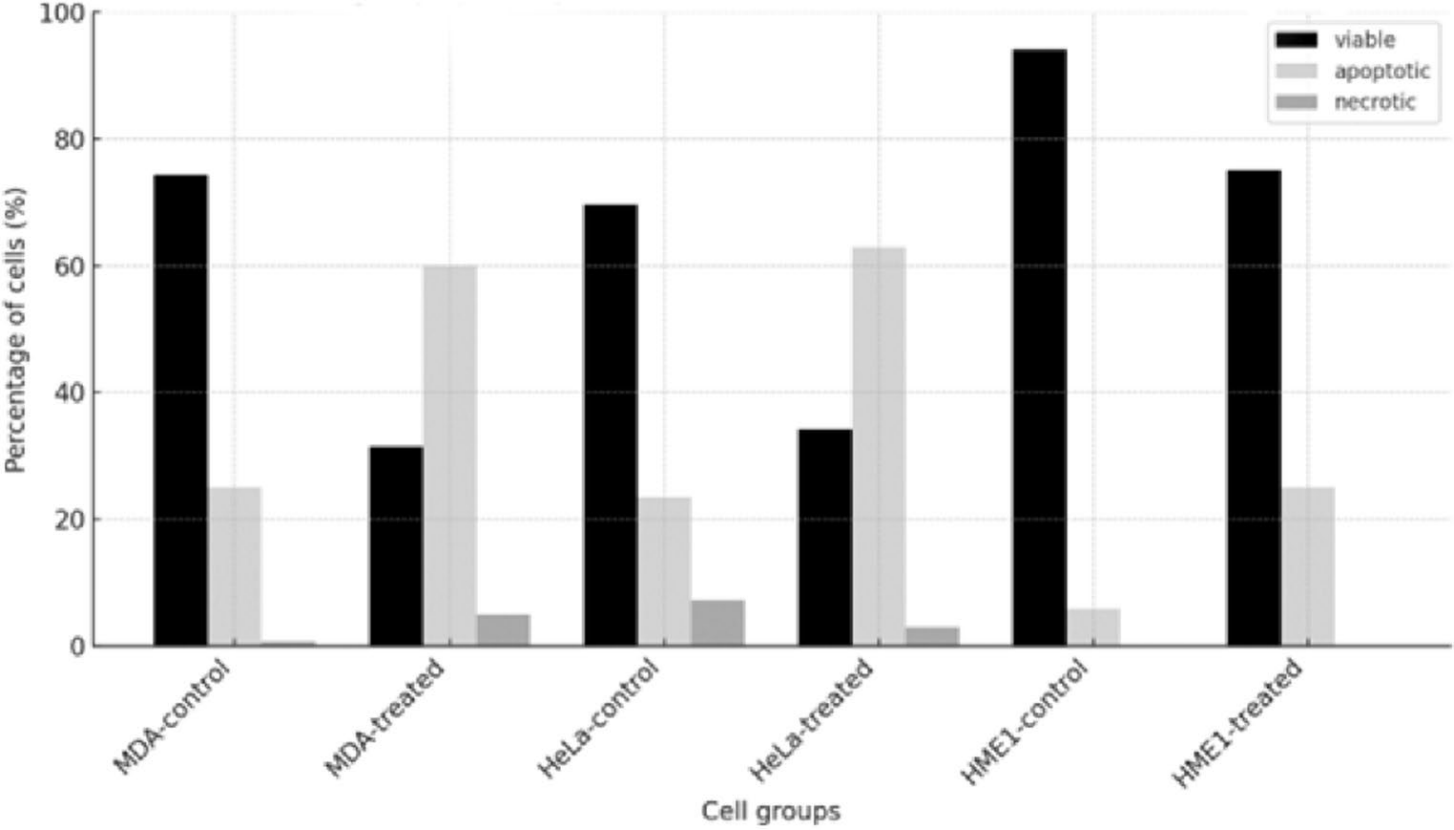
Apoptotic profiles of control and treated cells analyzed by fluorescence microscopy. The bar graph displays the percentages of viable, apoptotic, and necrotic cells in MDA-MB-231, HeLa, and HME1 cell lines under control and treated conditions. The black bars represent viable (live) cells, the light gray bars indicate apoptotic cells, and the dark gray bars represent necrotic cells. CS-CQDs treatment notably increased apoptosis (light gray) in MDA-MB-231 and HeLa cells, while viability (black) decreased. In contrast, normal HME1 cells showed minimal changes across all categories

Treatment with CS-CQDs markedly increased apoptosis in MDA-MB-231 cells from 25% to 55%, while the percentage of viable cells decreased from 74.3% to 40%, and necrosis slightly increased from 0.7% to 4.6%. In HeLa cells after treatment with CS-CQDs, apoptosis also increased from 24% to 65%, accompanied by a decrease in viability from 71% to 35%, and and a notable decrease in necrosis from 4.5% to 0%. HME1 non-cancerous cells showed only a mild change after treatment with CS-CQDs, with apoptosis increasing from 5.9% to 25% and viability decreasing from 94.1% to 75%, while no necrotic cells were observed in either condition (Fig. 10).

These findings indicate that CS-CQDs effectively induces apoptosis in cancer cell lines MDA-MB-231 and HeLa cells, while it does not induce apoptosis or necrosis in non-cancerous HME1 cells, suggesting selective cytotoxicity towards cancer cells. Importantly, the induction of apoptosis without accompanying necrosis highlights the selective and non-inflammatory cytotoxic nature of CS-CQDs, indicating that it may promote controlled cell death via apoptotic pathways rather than causing non-specific necrotic damage, which is a favorable characteristic for potential anti-cancer therapeutics.

Although no widely accepted morphology-based cytotoxicity assay currently exists due to the challenge of analyzing large numbers of samples, optical microscopy remains a practical method for morphological evaluation despite its limitations in distinguishing different types of cell death [31]. Therefore, cell morphology was examined to better understand the cytotoxic effects of CS-CQDs by comparing treated and untreated control groups in cancerous (HeLa and MDA-MB-231) and non-cancerous (hTERT-HME1) cell lines. Following treatment, significant cellular damage was observed in MDA-MB-231 and HeLa cells, consistent with the reduction in cell viability observed in the MTT assay. In contrast, although MTT results showed a 50% reduction in viability at the same IC₅₀ dose in non-cancerous hTERT-HME1 cells, their morphological integrity was largely preserved, suggesting that morphological deterioration does not always directly correlate with toxicity and that non-cancerous cells may be more resistant to CS-CQDs.

In the literature, apoptosis is considered a preferred cellular response in anti-cancer therapies as it enables cell death without triggering inflammation [32]. Consistently, CS-CQDS-induced apoptosis, without causing necrosis, supports its role as a controlled and targeted cytotoxic agent. Numerous plant-derived compounds have been reported to induce apoptosis in cancer cells while exerting minimal effects on normal cells [33], and similarly, CS-CQDs promotes selective apoptosis, enhancing its therapeutic potential. In this study, apoptosis analysis revealed that CS-CQDs significantly increased apoptotic rates in MDA-MB-231 (from 25% to 55%) and HeLa cells (from 24% to 65%), whereas the increase in apoptosis in non-cancerous HME1 cells remained limited (from 5.9% to 25%) with no necrosis observed. These findings indicate that CS-CQDs induces strong apoptotic effects in cancer cells while causing lower levels of cytotoxicity in normal cells, supporting its selective cytotoxic profile and potential safety as a therapeutic candidate [34].

In the literature, ZnO and doped ZnO nanoparticles synthesized using Psidium guajava leaf extract, particularly the GZC structure, have been reported to exhibit significant anticancer activity in the MOLT-4 cell line, with a low IC₅₀ value [35]. In the present study, CS-CQDs nanostructures demonstrated dose-dependent cytotoxic effects in different cell lines (MDA-MB-231 and HeLa), although the extent of this effect varied depending on the cell type.

Gene expression analysis further supported these findings. In MDA-MB-231 cells, although the absolute expression levels of both BAX and BCL-2 genes decreased compared to the control group, the BAX/BCL-2 ratio increased to 1.9, which is associated with the induction of apoptosis [36]. In HeLa cells, both BAX and BCL-2 expression levels were significantly increased; however, the BAX/BCL-2 ratio remained close to 1, suggesting a limited shift toward apoptosis. Nevertheless, considering the apoptosis assay results, a marked increase in apoptosis was observed, indicating that apoptosis may not solely depend on the BAX/BCL-2 ratio and that CS-CQDs may activate apoptotic pathways through alternative mechanisms [37].

The findings of the present study are partially consistent with previous reports demonstrating that natural extracts derived from edible mushrooms exhibit significant anticancer activity through the induction of apoptosis and inhibition of cell proliferation and migration in cervical cancer cells [38]. Similarly, CS-CQDs induced apoptosis and reduced cell viability in cancer cell lines, particularly in MDA-MB-231 and HeLa cells. The reported upregulation of pro-apoptotic markers such as p53, caspases, and the downregulation of Bcl-2 in mushroom extracts aligns with the pro-apoptotic effects observed in our study. However, while mushroom-derived extracts exert their effects primarily through bioactive phytochemicals with strong antioxidant properties, CS-CQDs represent a nanomaterial-based system that may induce cytotoxicity via distinct mechanisms, including oxidative stress modulation and differential regulation of NF-κB signaling.

Matrix metalloproteinases (MMPs) such as MMP2 and MMP9 facilitate extracellular matrix degradation and enhance tumor cell migration and invasion [39, 40]. In this study, a significant reduction in MMP2 and MMP9 gene expression was observed in both MDA-MB-231 and HeLa cells following CS-CQDs treatment, supporting its potential anti-metastatic effect. This finding is consistent with the reduced migration observed in MDA-MB-231 cells. However, in HeLa cells, despite the downregulation of MMP2 and MMP9, an increase in cell migration was observed, suggesting that CS-CQDs may influence migration through alternative mechanisms such as cytoskeletal remodeling or Laminin-5-related signaling pathways, highlighting the need for further mechanistic studies [41]. Additionally, CS-CQDs did not significantly affect migration in non-cancerous HME1 cells, indicating a more limited effect on normal cells and supporting its selective anti-cancer potential.

CS-CQDs treatment also significantly reduced the expression levels of HIF1A and IL-6 in both cancer cell lines. Given that HIF1A regulates hypoxia-related processes such as angiogenesis, metabolic adaptation, and drug resistance, and IL-6 is associated with inflammation, tumor growth, and metastasis, their downregulation suggests that CS-CQDs may suppress tumor progression by weakening tumor microenvironment-related processes [42].

The findings of the present study are partially consistent with those reported for *Cyclamen persicum* extract, which demonstrated significant antiproliferative, pro-apoptotic, and anti-migratory effects in MDA-MB-231 cells [43]. Similarly, CS-CQDs induced a marked reduction in cell viability and promoted apoptosis, particularly in cancer cells. However, unlike *C. persicum*, which exhibited clear inhibition of migration and invasion, CS-CQDs showed a more variable, cell-type-dependent effect on migration, with a pronounced decrease observed only in MDA-MB-231 cells. Moreover, while *C. persicum* extract suppressed NF-κB signaling via reduced p65 phosphorylation, CS-CQDs treatment was associated with increased NF-κB expression, suggesting distinct underlying molecular mechanisms.

Although both studies involve modulation of NF-κB signaling, their biological outcomes differ significantly. Piperine has been reported to exert photoprotective effects by reducing ROS generation, preventing DNA damage, and suppressing NF-κB activation via upregulation of IκB-α, thereby promoting cell survival [44]. In contrast, CS-CQDs in the present study induced cytotoxicity and apoptosis in cancer cells, accompanied by increased NF-κB expression. These findings highlight the context-dependent role of NF-κB signaling, acting as a survival pathway in normal cells under stress conditions while contributing to pro-apoptotic responses in cancer cells. The observed differences may stem from the distinct biological properties of phytochemical compounds and nanomaterial-based systems.

Furthermore, NF-κB expression was significantly increased in both MDA-MB-231 and HeLa cells following CS-CQDs treatment. Although NF-κB is commonly associated with tumor progression and inflammation, it is also activated in response to cellular stress [31]. Therefore, its upregulation may reflect a stress response induced by oxidative or endoplasmic reticulum stress rather than a pro-tumorigenic effect. Additionally, certain natural compounds have been reported to transiently activate NF-κB signaling while promoting apoptosis [45]. In this context, the increased NF-κB expression observed alongside apoptosis may represent a temporary stress-related activation, suggesting that CS-CQDs may exert both regulatory and stress-mediated effects. However, further studies investigating NF-κB target genes, phosphorylation status, and nuclear translocation are required to fully elucidate this mechanism.

## Conclusions

Carbon nanodots synthesized from *Chaenomeles speciosa* flowers via hydrothermal carbonization are characterized as a heterogeneous nanomaterial with a predominantly amorphous structure and weak crystallinity, as confirmed by XRD, FTIR, XPS, and HRTEM analyses. The structure consists mainly of an amorphous carbon framework, limited crystalline regions (lattice spacing of 0.093–0.091 nm), a high content of oxygen and nitrogen functional groups (C: 68.28%, O: 23.03%, N: 6.70%), and a heteroatom (O, N)-rich surface. The natural phenolic compounds and amino acids present in the plant material enable effective heteroatom integration. Biological evaluations showed that CS-CQDs exert dose-dependent cytotoxic effects in cancer cell lines (MDA-MB-231 and HeLa) while inducing minimal toxicity in non-cancerous HME-1 cells. Morphological observations and gene expression analyses indicated apoptosis as the primary mechanism of cell death in cancer cells, with selective activation of apoptotic pathways and modulation of NF-κB signaling. Furthermore, CS-CQDs reduced migration in highly motile MDA-MB-231 cells, suggesting potential anti-metastatic effects, whereas non-cancerous cells were largely unaffected. Overall, CS-CQDs exhibits selective cytotoxicity toward cancer cells through apoptotic induction without triggering necrosis in healthy cells, highlighting their potential as a biocompatible and targeted anti-cancer nanomaterial. These findings provide a promising basis for further development of plant-derived carbon nanodots in cancer therapeutics. 

## Electronic Supplementary Material

Below is the link to the electronic supplementary material.

Supplementary Material 1

## Data Availability

Not applicable.

## Declarations

### Artificial Intelligence Statement

Artificial intelligence-based tools were used solely for language editing and translation purposes to improve the clarity and readability of the manuscript. No AI tools were used in data analysis, interpretation of results, or generation of scientific content.
