# Supplementary figures and images for: Green-Synthesized Chaenomeles speciosa–Derived Carbon Quantum Dots with Blue Fluorescence and Selective Pro-Apoptotic Effects in Cancer Cells
Source: J Fluoresc. 2026 Apr 29;36(5):3451–65. doi: 10.1007/s10895-026-04771-y (PMC13226346; doi:10.1007/s10895-026-04771-y)

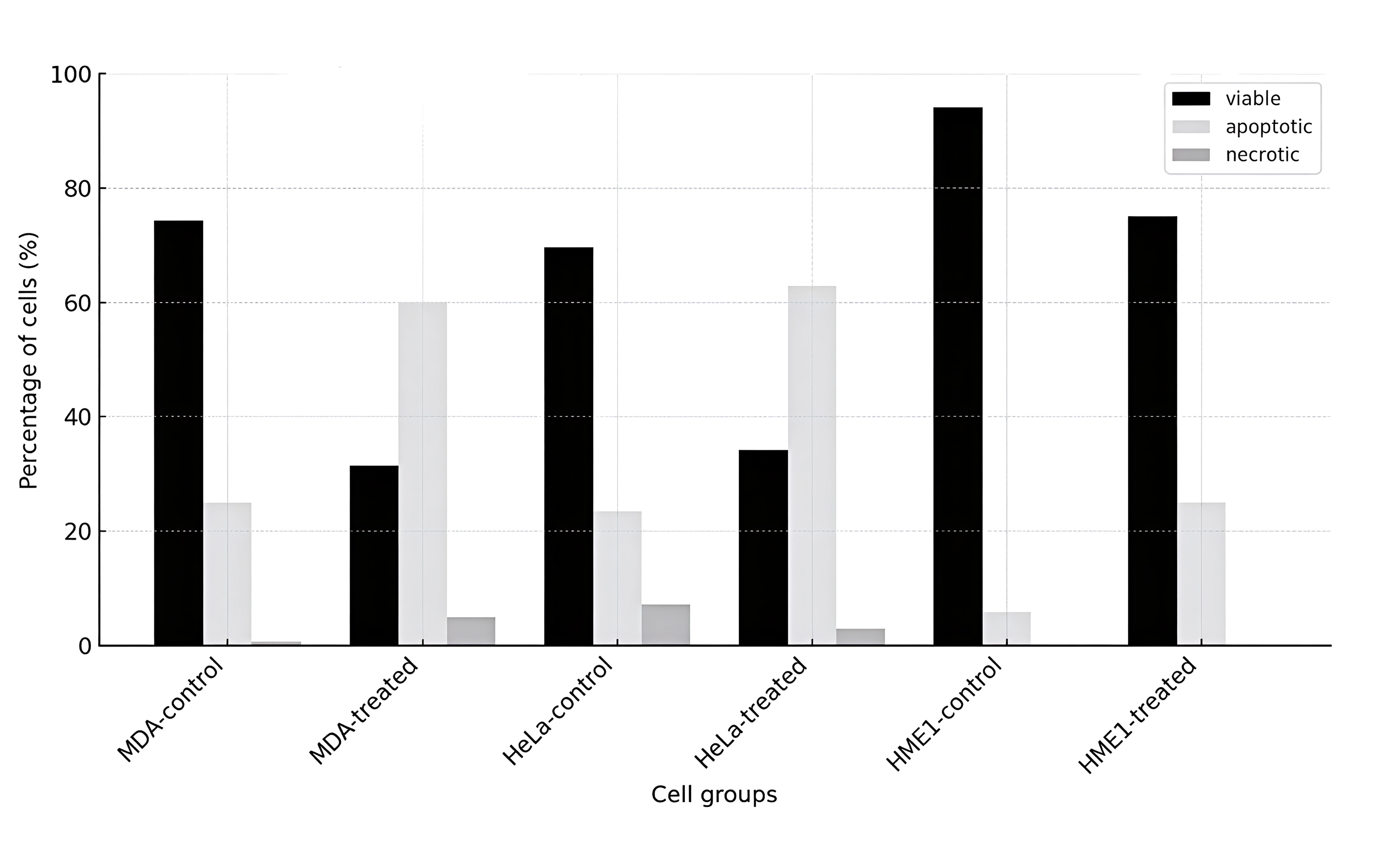

Supplement: Supplementary file 1 — Supplementary Material 1 [file 10895_2026_4771_MOESM1_ESM.tiff]
